# Differential expression profiling of the early response to *Ustilaginoidea virens* between false smut resistant and susceptible rice varieties

**DOI:** 10.1186/s12864-015-2193-x

**Published:** 2015-11-16

**Authors:** Yanqing Han, Kang Zhang, Jun Yang, Nan Zhang, Anfei Fang, Yong Zhang, Yongfeng Liu, Zhiyi Chen, Tom Hsiang, Wenxian Sun

**Affiliations:** Department of Plant Pathology, China Agricultural University, 2 West Yuanmingyuan Rd., Haidian District, Beijing, 100193 China; Key Laboratory of Plant Pathology, Ministry of Agriculture, China Agricultural University, Beijing, 100193 China; Institute of Plant Protection, Jiangsu Academy of Agricultural Sciences, Nanjing, 210014 China; School of Environmental Sciences, University of Guelph, Guelph, ON N1G 2 W1 Canada

**Keywords:** Differential expression profiling, Protein kinases, *Pathogenesis-related* genes, Phytoalexins, Resistance, Rice false smut, *Ustilaginoidea virens*

## Abstract

**Background:**

Rice false smut caused by *Ustilaginoidea virens* has recently become one of the most devastating rice diseases worldwide. Breeding and deployment of resistant varieties is considered as the most effective strategy to control this disease. However, little is known about the genes and molecular mechanisms underlying rice resistance against *U. virens*.

**Results:**

To explore genetic basis of rice resistance to *U. virens*, differential expression profiles in resistant ‘IR28’ and susceptible ‘LYP9’ cultivars during early stages of *U. virens* infection were compared using RNA-Seq data. The analyses revealed that 748 genes were up-regulated only in the resistant variety and 438 genes showed opposite expression patterns between the two genotypes. The genes encoding receptor-like kinases and cytoplasmic kinases were highly enriched in this pool of oppositely expressed genes. Many pathogenesis-related (*PR*) and diterpene phytoalexin biosynthetic genes were specifically induced in the resistant variety. Interestingly, the RY repeat motif was significantly more abundant in the 5’-regulatory regions of these differentially regulated *PR* genes. Several WRKY transcription factors were also differentially regulated in the two genotypes, which is consistent with our finding that the *cis*-regulatory W-boxes were abundant in the promoter regions of up-regulated genes in IR28. Furthermore, *U. virens* genes that are relevant to fungal reproduction and pathogenicity were found to be suppressed in the resistant cultivar.

**Conclusion:**

Our results indicate that rice resistance to false smut may be attributable to plant perception of pathogen-associated molecular patterns, activation of resistance signaling pathways, induced production of PR proteins and diterpene phytoalexins, and suppression of pathogenicity genes in *U. virens* as well.

**Electronic supplementary material:**

The online version of this article (doi:10.1186/s12864-015-2193-x) contains supplementary material, which is available to authorized users.

## Background

Rice false smut (RFS) caused by the Clavicipitaceous fungus *Ustilaginoidea virens*, also known as *Villosiclava virens*, has recently become one of the most devastating grain diseases in the majority of rice-planting regions worldwide [1]. RFS was first reported in Tirunelveli district of Tamil Nadu State of India and previously categorized as a minor disease due to its sporadic occurrence [2]. However, the disease has expanded rapidly in China due to large-scale planting of high-yield rice cultivars and hybrids, heavy application of nitrogenous fertilizer and global warming in the past two decades, and has been found in about one third of rice cultivation areas in severe years [1, 3]. RFS outbreaks have also been reported in some American, Italian and Southern Asian rice-growing regions [4]. The disease incidence rate was estimated to be 15.85 % in 2011 across northern India, and the smut balls formed on up to 100 grains per panicle in some fields with high disease severity [5].

Aside from huge yield losses (up to 40 % in severe years) caused by RFS, *U. virens* produces abundant amounts of mycotoxins that often contaminate rice products and are poisonous to both human and animals [6–8]. Due to the economic importance of the disease, many studies have been performed on the occurrence, pathogen detection, mycotoxin identification, infection lifecycle and chemical control of the disease [4, 9–12]. However, research on screening of rice germplasm for RFS resistance, molecular mechanisms underlying RFS resistance and the pathogenicity of *U. virens* is scarce [13]. Breeding for rice cultivars with durable resistance to RFS is considered to be one of the most economical, environmentally safe and effective strategies for disease management. A rapid and effective inoculation method has been developed to evaluate rice resistance to *U. virens* and screen resistant germplasm for breeding [14, 15]. Although no rice variety has yet been identified to have complete or high level of resistance, cultivars do exhibit significant differences in quantitative resistance to *U. virens* [16, 17]. Much effort has been taken to identify quantitative trait loci (QTL) associated with rice resistance to *U. virens* [17–19]*.* It was reported that the rice cultivar IR28 has a relatively high resistance to RFS, which was controlled by two major and multiple minor resistance genes [17]. Eight QTLs controlling RFS resistance were also found in the resistant rice variety Lemont [19]. However, no QTL for RFS resistance in rice has yet been isolated and resistance mechanisms are largely unknown [17].

In plants, multiple strategies have evolved to recognize pathogens and thus trigger immune systems to defend against pathogen invasion. Recognition of conserved pathogen-associated molecular patterns (PAMPs) by pattern recognition receptors (PRRs) activates PAMP-triggered immunity (PTI) and prevents further colonization on the hosts by microbial pathogens [20]. Perception of pathogen effectors by intercellular R proteins in plants activates effector-triggered immunity (ETI), which includes rapid and acute cell death responses in plants and restricts multiplication of pathogens [21]. Furthermore, systemic acquired resistance (SAR) induced by the signal molecule salicylic acid (SA) may confer long-lasting protection against a wide range of pathogens [22].

Pathogenesis-related (*PR*) genes are often induced in plant defense signaling through the action of plant hormones including salicylic acid, jasmonic acid or ethylene [23]. In *Arabidopsis*, expression of *PR1*, *PR2* and *PR5* is induced by SA and used as a signature for SAR [24]. These induced PR proteins possess antimicrobial activities through their hydrolytic, proteinase-inhibitory and membrane-permeabilizing abilities, or serve as defense signals [22, 23]. As an example, PR-2 proteins function as β-1,3-glucanases that catalyze the hydrolytic cleavage of 1,3-β-D-glucosidic linkages in β-1,3-glucans present in the fungal cell walls. The disrupted cell walls cause cell lysis and death in fungi [25]. The PR-3 proteins possess endo-chitinase activities and retard fungal growth by the enzymatic hydrolysis of chitin, the predominant constituent of fungal cell walls. The released chitin fragments often act as endogenous triggers to stimulate plant defenses [26]. Peroxidases (PR-9) are heme-containing glycoproteins that participate in a number of physiological processes, such as biosynthesis of ethylene, suberization and lignification of plant cells in response to pathogen infection, wounding and abiotic stresses [27, 28].

Comprehensive transcriptome analyses during the interaction of plants and pathogens are commonly used to provide new insights into molecular mechanisms of plant resistance. Transcriptome comparisons between durable resistant and susceptible rice varieties in response to attack by the blast fungus *Magnaporthe oryzae* revealed that chitin-oligosaccharide sensing factors, wall-associated kinases, MAPK cascades and WRKY transcription factors were involved in rice blast resistance [29]. In addition, gene expression profiling of rice in response to the infection of rice stripe virus (RSV) and small brown plant-hopper (SBPH) revealed by transcriptome analyses indicated that the jasmonate signaling pathway was important in rice resistance to SBPHs [30]. Transcriptome analyses were also performed for other host-pathogen interaction through RNA-Seq, including wheat and *Fusarium graminearum* [31], maize and *Sporisorium reilianum* f. sp. *zeae* [32], cotton and the wilt fungus *Verticillium dahliae* [33], soybean and *Xanthomonas axonopodis* pv. *glycines* [34], banana and *F. oxysporum* f. sp. *cubense* [35]. Many genes were thereby revealed to be involved in resistance-associated signal transduction and defense mechanism in plants. For example, *PR* genes were found to be significantly up-regulated in rice after blast fungus inoculation [36] and in the maize resistant variety Mo17 in response to *S. reilianum* f. sp. *zeae* [32].

Recently, RNA-Seq has been used to reveal stage-specific biological processes related to the compatible rice-*U. virens* interaction and expression profiling in rice varieties at the late stage of *U. virens* infection [37, 38]. It was reported that the primary site of *U. virens* colonization was at the base of the filaments with the inner spikelets becoming infected by hyphae at 24 h post inoculation (hpi) [39]. Here, we analyzed and compared gene expression profiles of the RFS resistant variety IR28 and susceptible LYP9 after *U. virens* inoculation at early stages (24 hpi and 48 hpi) using transcriptome data. The results indicate that several major gene families might be involved in rice resistance to *U. virens* infection, including receptor-like kinases, *PR* genes, diterpene phytoalexin biosynthesis genes and WRKY transcription factors. These results provide important information to further understand molecular mechanisms of rice reaction and resistance to false smut.

## Results

### Disease symptoms of false smut in rice cultivars IR28 and LYP9

To confirm RFS resistance or susceptibility of IR28 and LYP9, disease symptoms were observed on the panicles inoculated with different *U. virens* isolates. Infected grains per inoculated spikelet of the cultivars IR28 and LYP9 were counted (Table 1 and Additional file 1: Figure S1). In general, more false smut balls were produced on LYP9 panicles than those on IR28 panicles for each of three isolates. The average number of false smut balls per panicle formed on LYP9 (26.2 ± 2.40) was significantly more than that on IR28 (5.75 ± 0.74) after P1 inoculation. The number of false smut balls produced on both cultivars inoculated with 37–1 and 39–3 was less than that formed on the panicles after P1 inoculation. These data confirm that the cultivar IR28 is much more resistant to *U. virens* than LYP9. The results also indicate that virulence to both rice cultivars of the isolates 37–1 and 39–3 is much less than that of P1. Therefore, the P1 isolate was chosen for inoculation in further expression profiling analyses.

**Table 1:** Virulence assays of three *U. virens* isolates (37–1, 39–3 and P1) to the varieties IR28 and LYP9, showing that IR28 is significantly more resistant to *U. virens* infection than LYP9

Isolates	Infected panicle rate		False smut balls per panicle		*P*_value
	IR28	LYP9	IR28	LYP9	
Mock	0 % (*n* = 20)	0 % (*n* = 20)	0	0	
37–1	20 % (*n* = 20)	50 % (*n* = 20)	0.45 ± 0.23	1.05 ± 0.30	0.03525
39–3	90 % (*n* = 20)	95 % (*n* = 20)	1.05 ± 0.20	4.80 ± 0.65	3.34E-05
P1	100 % (*n* = 20)	100 % (*n* = 15)	5.75 ± 0.74	26.2 ± 2.40	1.43E-07

*n* stands for the number of panicles

### RNA-Seq data and aligning to the reference genomes

Changes in gene expression level of rice cultivars IR28 and LYP9 at 24 h and 48 h after P1 inoculation were analyzed using RNA-Seq data. A total of 64.4 million clean reads, each of which was 49 bp in length, were generated from eight cDNA libraries (the susceptible cultivar LYP9 and resistant cultivar IR28 at 24 and 48 hpi and four mock-inoculated controls). About 82 % of the clean reads were successfully aligned to the *Oryza sativa* L. spp. *indica* reference genome (Additional file 2: Table S1). Saturation analysis showed that newly emerging tags were gradually reduced as the total number of sequence tags increased, and the detectable tags approached saturation when the number of sequencing tags reached ~3 million (Additional file 3: Figure S2). These results indicate that the gene transcript data were reliable, and suitable for further transcriptome analysis.

### Expression profiling analyses in resistant and susceptible cultivars in response to *U. virens* inoculation

**Fig. 1:**
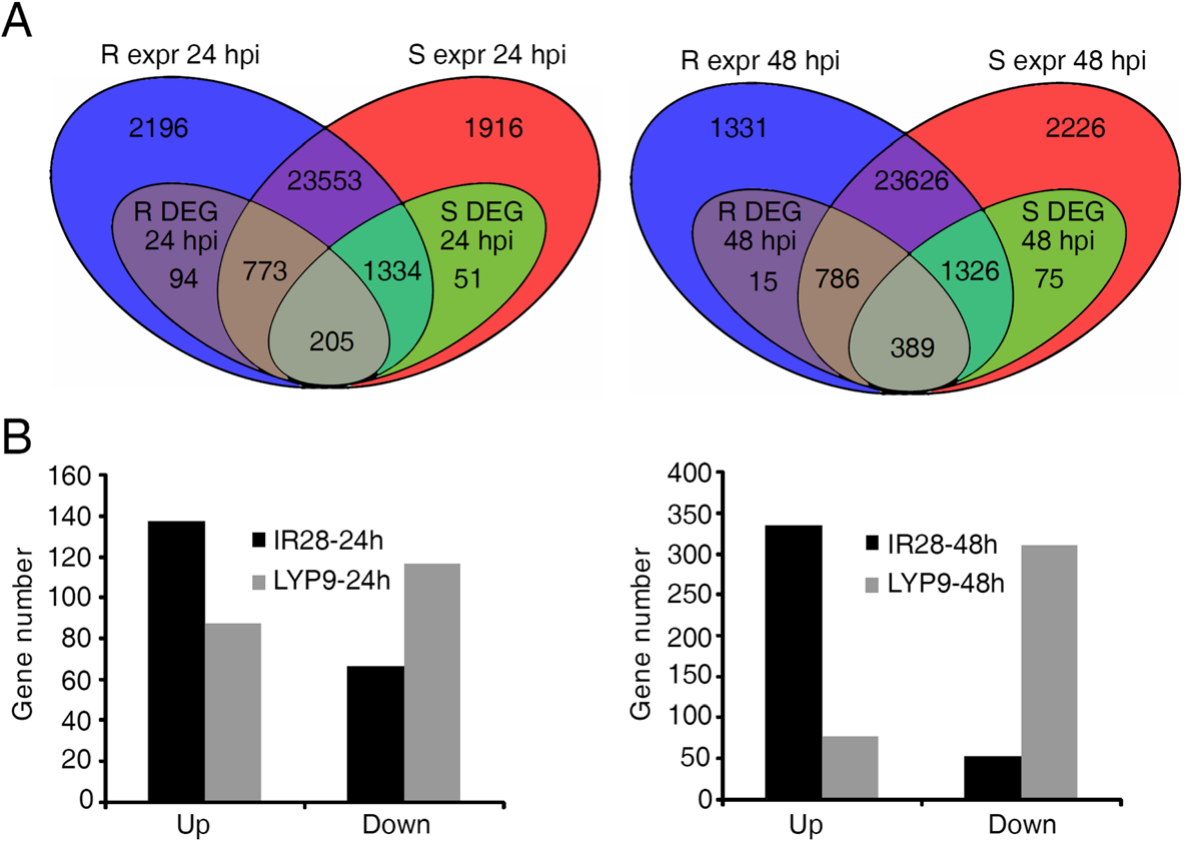
Venn diagrams of all differentially expressed genes in the resistant variety IR28 and susceptible variety LYP9 in the early stages of *U. virens* infection. **a** The expressed genes (expr) and differentially expressed genes (DEGs) in IR28 (R) and LYP9 (S) at 24 hpi and 48 hpi. A total of 205 DEGs were common in IR28 and LYP9 at 24 hpi while 389 DEGs were common at 48 hpi. **b** Up-regulated and down-regulated genes among common DEGs in IR28 and LYP9 at 24 hpi and 48 hpi. Among common DEGs, more DEGs were up-regulated in IR28 while more DEGs were down-regulated in LYP9

To uncover the genes that might be involved in RFS resistance, all differentially expressed genes (DEGs) were identified in IR28 and LYP9 at 24 h and 48 h after P1 inoculation as compared with mock-inoculated samples. Venn diagrams were then drawn to show DEGs that were common to both rice genotypes IR28 and LYP9, or specific to either cultivar in response to P1 inoculation (Fig. 1a). A total of 1072 DEGs were identified in IR28 at 24 hpi, among which 94 were IR28-specific, 205 were common to IR28 and LYP9, and 773 were DEGs only in IR28 but were expressed (non-DEG) in LYP9. In contrast, 1590 DEGs were identified in LYP9 including 51 LYP9-specific and 1334 were DEGs only in LYP9 but expressed in IR28. Meanwhile, 1190 and 1790 DEGs were identified in IR28 and LYP9 at 48 hpi, respectively. Among them, 389 were identified as DEGs common to IR28 and LYP9, and 786 were categorized as DEGs only in IR28 but expressed in LYP9; 75 were LYP9-specific, and 1326 were found as DEGs only in LYP9 but expressed in IR28. Among common DEGs shared by both cultivars at 24 hpi, more genes were up-regulated in IR28 (138) than in LYP9 (88) while fewer DEGs (67) in IR28 were down-regulated than those (117) in LYP9. Among 389 common DEGs at 48 hpi, more (335) were up-regulated compared with down-regulated ones (54) in IR28, while many more genes (311) were suppressed than transcriptionally induced genes (78) in LYP9 at this infection stage (Fig. 1b). Interestingly, the majority of common DEGs (438) exhibited opposite expression patterns between the resistant and susceptible varieties after P1 inoculation, suggesting that defense responses are distinctively different between the two varieties in response to *U. virens* infection. We speculate that the IR28-specific DEGs and common DEGs which were up-regulated in IR28 and down-regulated in LYP9 might be involving in RFS resistance (Additional file 4: Table S2).

### Comparison between transcriptomes of IR28 and LYP9 in response to *U. virens* infection by cluster analysis

**Fig. 2:**
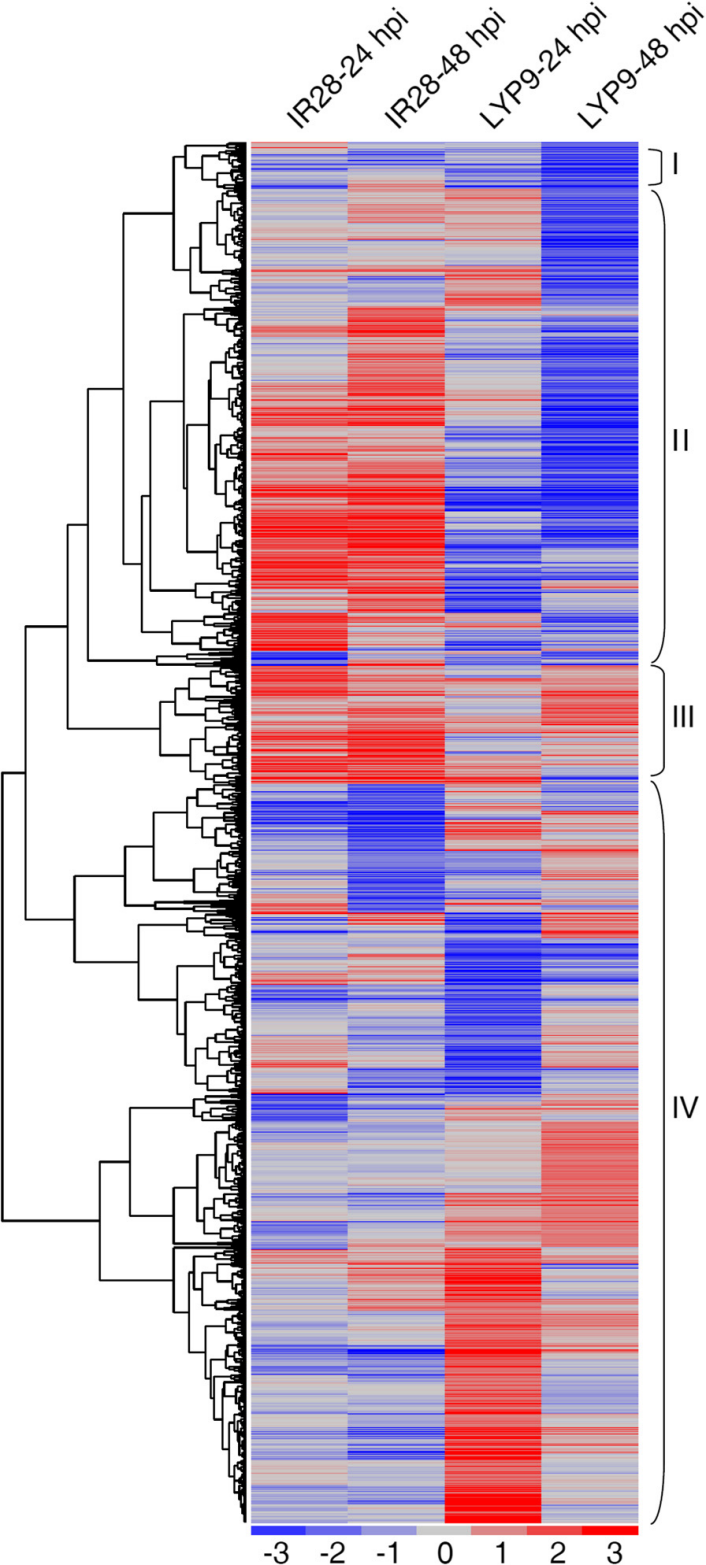
The expression pattern of differentially regulated genes in IR28 and LYP9 during the early stages of *U. virens* infection. A total of 3847 genes were identified to be differentially regulated in IR28 and LYP9 in response to *U. virens* at 24 hpi and 48 hpi. Each column represents the Log_2_ fold change in transcript levels in rice at the indicated times, relative to the levels of mock-inoculated samples. The vertical dimension represents the genes that exhibited changes in transcript level (cutoff: |log_2_[fold change]| ≥ 1 and FDR ≤ 0.001). The colour scale indicates transcript abundance relative to the mock-inoculated panicles: red, increase in relative transcript abundance; blue, decrease in relative transcript abundance

A total of 3847 DEGs in IR28 and in LYP9 were classified through cluster analysis. The heat map generated by cluster analysis showed that the majority of DEGs have similar expression patterns between two different time points in the same cultivar. The analysis also showed that these DEGs can be categorized into four major groups: genes down-regulated in both IR28 and LYP9 (group I); genes up-regulated in IR28 while down-regulated in LYP9 (group II); genes up-regulated in both IR28 and LYP9 (group III); and genes up-regulated in LYP9 while suppressed in IR28 (group IV) (Fig. 2). It was speculated that the genes specifically up-regulated in IR28 may play important roles in RFS resistance.

### Gene ontology enrichment analysis

To investigate functions or biological processes that the differentially regulated genes might be involved in, gene ontology (GO) enrichment analysis was performed to classify up-regulated DEGs (Additional file 5: Table S3). Within three major GO categories (cellular components, molecular functions and biological processes), 14 common GO terms, 2 IR28-specific and 31 LYP9-specific GO terms were enriched at 24 hpi, while 12 common GO, 1 IR28-specific and 30 LYP9-specific GO terms were enriched at 48 hpi. The gene names in the GO terms enriched specifically by IR28 were searched for items that might be related to RFS resistance. Among them, the GO term “transferase activity” was the only one that was significantly enriched (*P* ≤ 0.05) in IR28 at both inoculation time points. It is most likely that some genes with transferase activity are involved in RFS resistance (Additional file 6: Figure S3).

### Some protein kinases including receptor-like kinases are likely involved in RFS resistance

**Fig. 3:**
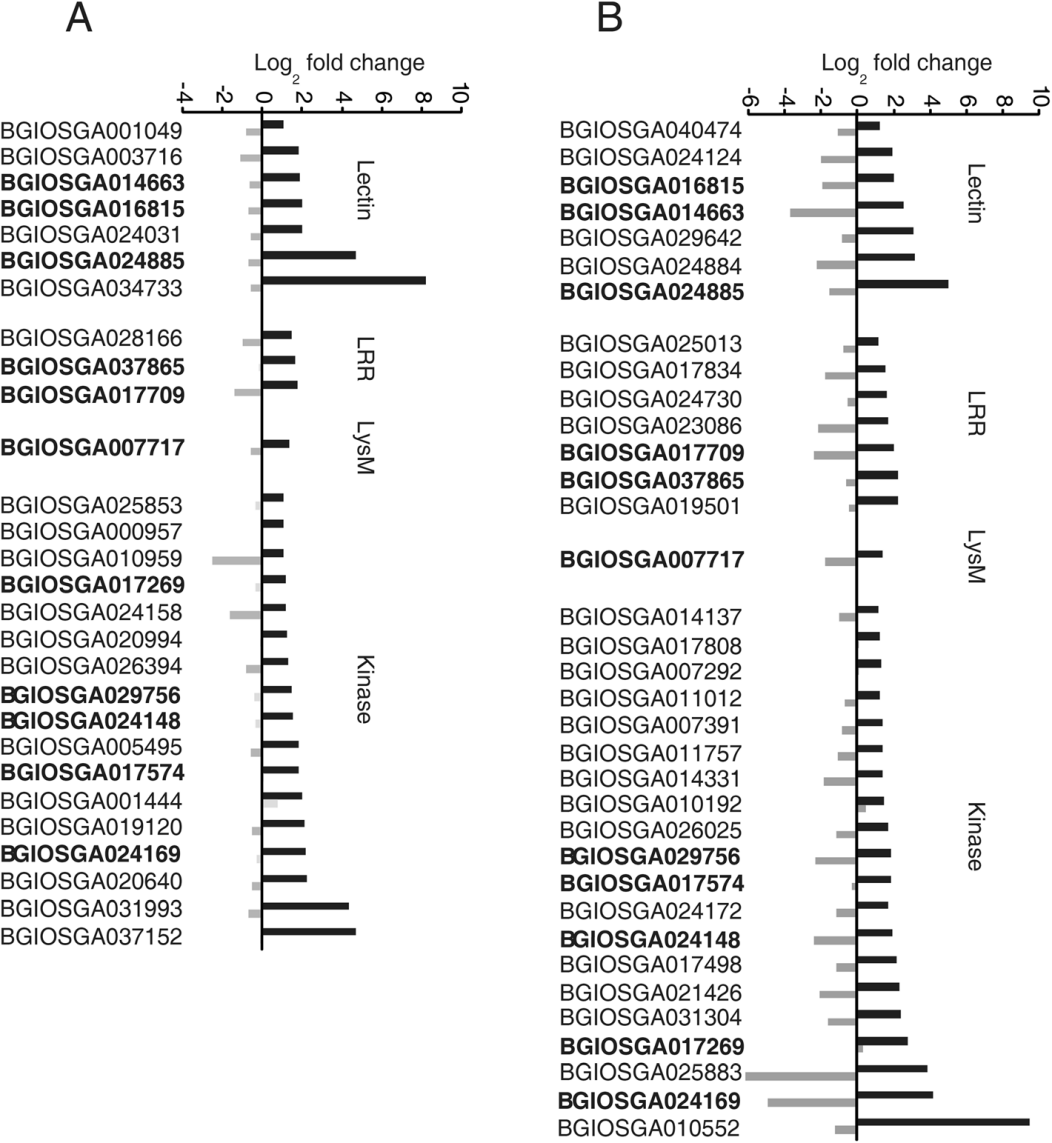
The protein kinase genes exhibiting opposite expression patterns between IR28 and LYP9 in response to *U. virens* infection. A total of 28 and 35 protein kinase genes were identified to have opposite expression patterns between the two genotypes at 24 hpi (**a**) and 48 hpi (**b**). Bold fonts indicate the protein kinase genes that have a consistent expression pattern between two inoculation time points. Lectin, LRR, LysM and kinase indicate lectin-receptor like kinases, leucine-rich repeat containing receptor-like kinases and lysin motif-containing receptor-like kinases and cytoplasmic kinases, respectively

The 142 DEGs in IR28 that were categorized into the GO term “transferase activity” were subject to Pfam domain searches. The results showed that the majority of these DEGs belonged to two gene families encoding protein kinases and glutathione S transferases (Additional file 7: Table S4). Comparison of gene expression levels between the resistant and susceptible cultivars showed that expression of glutathione S transferases was not significantly different between IR28 and LYP9. In contrast, the differentially-regulated protein kinases exhibited distinctive expression patterns between the two cultivars (Additional file 6: Figure S3). Twenty-eight and 35 protein kinase genes were induced in IR28 at 24 hpi and 48 hpi, respectively (Fig. 3). Notably, 11 protein kinase genes were up-regulated at both inoculation time points. All of the induced protein kinase genes in IR28 at 24 hpi except BGIOSGA00144 were transcriptionally suppressed in LYP9. Meanwhile, the up-regulated kinase genes at 48 hpi in IR28 except BGIOSGA010192 and BGIOSGA017269 were also down-regulated in LYP9 (Fig. 3). These differentially regulated kinases were classified into four groups, B-lectin receptor-like kinases, leucine-rich repeat (LRR) receptor-like kinases, LysM domain-containing receptor kinases and cytoplasmic kinases (Fig. 3). In particular, a B-lectin receptor kinase gene (BGIOSGA024885) was dramatically induced at both time points. Another B-lectin receptor kinase gene (BGIOSGA034733) and a protein kinase gene (BGIOSGA010552) were found to be greatly up-regulated with 239- and 306-fold expression in IR28 at 24 hpi and 48 hpi, respectively. Divergent patterns of expression in the resistant and susceptible cultivars strongly suggest that these kinase-encoding genes might be essential for rice resistance to RFS in IR28.

### Expression profiles of pathogenesis-related genes

**Fig. 4:**
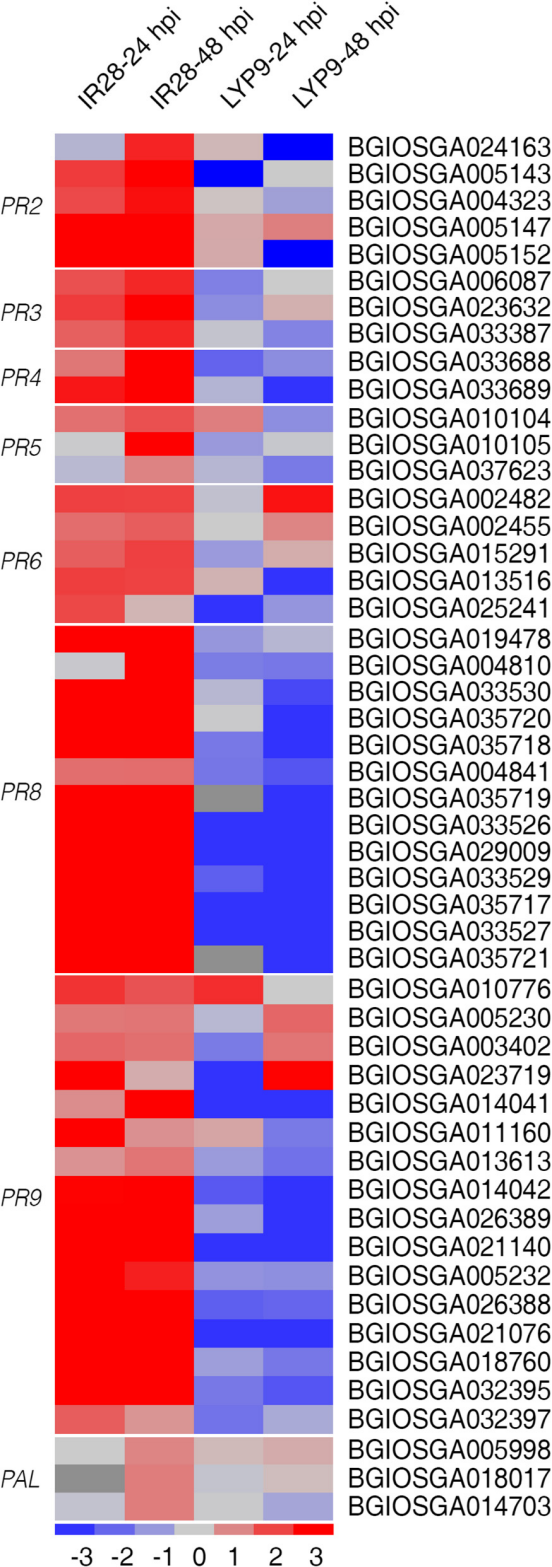
Heat maps showing expression patterns of pathogenesis-related and *PAL* genes that were identified to be differentially regulated in IR28 and LYP9. The technical details and the colour scale are the same as those in Fig. 2

To identify other important genes that might be involved in biosynthetic or signaling pathways critical for RFS resistance in IR28, pathway enrichment analyses were performed using KEGG (Additional file 8: Table S5). Only a few defense-associated biosynthetic pathways involving diterpenoid, cutin, suberine or wax were enriched in the transcriptome of the resistant cultivar, while more pathways, such as phenylalanine metabolism and secondary metabolite biosynthesis, were significantly enriched in both rice genotypes after inoculation. Comparisons of expression levels of DEGs in these enriched pathways revealed that many genes were up-regulated in the resistant cultivar, while down-regulated in the susceptible LYP9. Among them, multiple gene families encoding PR proteins were greatly up-regulated after P1 inoculation in IR28 (Fig. 4).

Five β-1,3-glucanase genes belonging to the *PR2* family exhibited significantly different expression patterns between IR28 and LYP9 after P1 inoculation (Additional file 9: Table S6). In IR28, these genes were transcriptionally induced at 24 hpi and up-regulated even more dramatically at 48 hpi. In contrast, these genes were generally suppressed or not significantly regulated at both time points in LYP9. Extensive transcriptome analyses in both cultivars also showed that three class I (*PR3*), two class II (*PR4*) and 13 class III chitinase genes (*PR8*) were up-regulated at 24 hpi and 48 hpi in IR28, while these genes were generally down-regulated at the two time points in LYP9 (Fig. 4 and Additional file 9: Table S6). It is interesting to note that genes BGIOSGA035717 to 21, BGIOSGA033526, BGIOSGA033527, BGIOSGA033529 and BGIOSGA033530 were tandemly arranged in a chitinase gene cluster on chromosome 11. In addition, 16 peroxidase genes (*PR9*), 3 thaumatin-like genes (*PR5*) and 5 proteinase inhibitor genes (*PR6*) were identified as being induced in IR28 while most were inhibited in LYP9 after P1 inoculation. Phenylalanine ammonia-lyases (PALs), sometimes classified as PR proteins, are involved in the synthesis of both phytoalexins and lignin, to inhibit pathogens from penetrating cell walls [40]. Three *PAL* genes (BGIOSGA014703, BGIOSGA018017 and BGIOSGA005998) involved in the phenylalanine metabolism and phenylpropanoid biosynthesis pathways were also up-regulated only in IR28 (Fig. 4 and Additional file 9: Table S6). Taken together, our finding that many defense-related genes including *PR* and *PAL* genes showed opposite expression patterns between IR28 and LYP9 after *U. virens* inoculation indicates that these genes play essential roles in RFS resistance in IR28.

### Diterpene phytoalexin biosynthesis genes

**Fig. 5:**
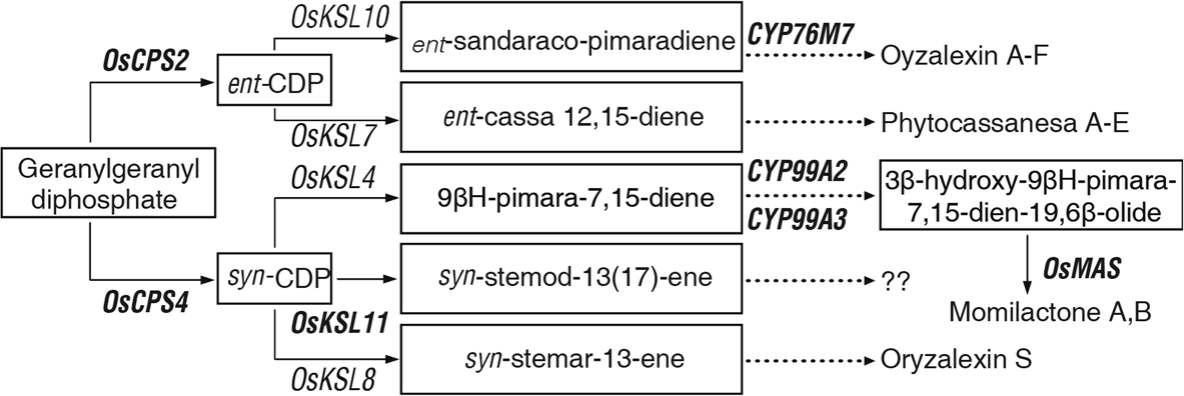
The genes involved in phytoalexin biosynthesis were specifically induced in the resistant variety IR28 in response to *U. virens* infection. Known genes that are responsible for the biosynthesis of different types of phytoalexins were shown. Genes in bold were identified to be up-regulated in IR28

A total of 15 phytoalexins (PAs) have been characterized in rice, including 14 diterpenoid PAs and one flavonoid PA, sakuranetin [41, 42]. The diterpenoid PAs in rice have been categorized into four distinct types: phytocassanes A to E, oryzalexins A to F, momilactones A and B, and oryzalexin S [43]. Many essential genes involved in phytoalexin biosynthesis pathways were previously identified (Fig. 5). Among them, seven genes were significantly up-regulated in IR28 at 24 hpi and 48 hpi, and enriched specifically in DEGs of the resistant cultivar revealed by KEGG pathway enrichment analyses. These genes included *OsCPS*2 (BGIOSGA008469) which is involved in the phytocassane A-E synthesis, *OsCPS4* (BGIOSGA015502), *CYP99A2* (BGIOSGA015504), *CYP99A3* (BGIOSGA015981), and *OsMAS* (BGIOSGA038038) which are involved in the biosynthesis of momilactone A and B, *CYP76M7* (BGIOSGA008466) that catalyzes the biosynthesis of oyzalexin A-F, and *OsKSL11* (BGIOSGA034012) (Fig. 5 and Additional file 10: Table S7). Up-regulation of these phytoalexin biosynthesis genes in response to *U. virens* indicates that phytoalexins are important components in rice resistance to RFS.

### Differential expression of WRKY transcription factors

**Fig. 6:**
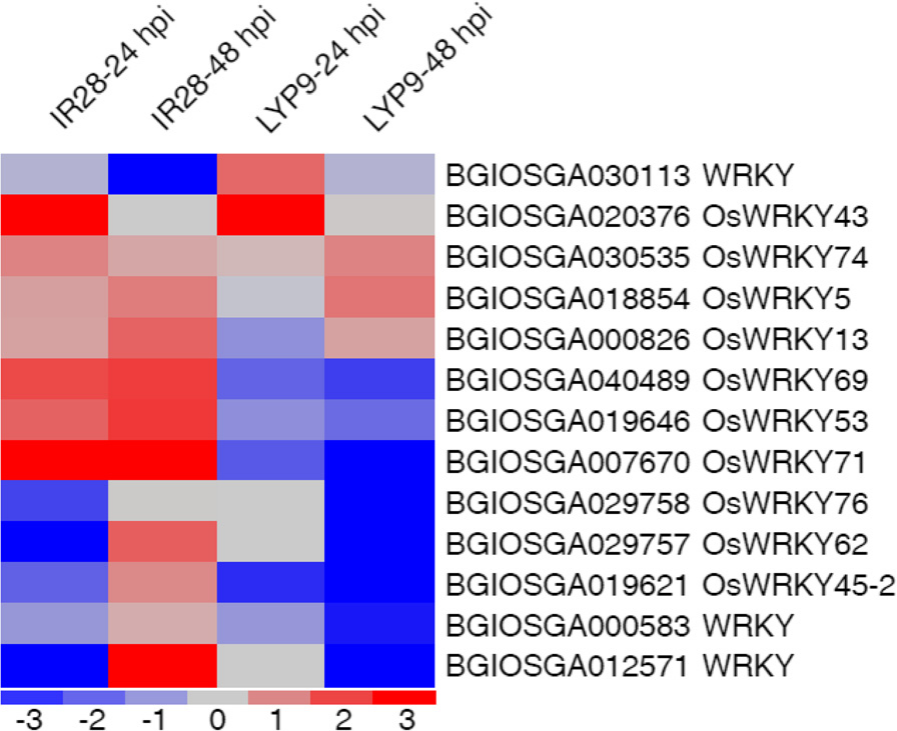
Heat map for differentially-regulated WRKY genes between IR28 and LYP9. A total of 13 WRKY genes were identified to be differentially regulated in IR28 and LYP9. The technical details and the colour scale are the same as those in Fig. 2

WRKY transcription factors are one of the largest protein superfamilies in plants that can regulate various defense processes and play important roles in controlling the transcription of defense-related genes through binding to W-boxes in their promoters, a key *cis*-element in defense-related transcriptional regulation [44, 45]. Here, we identified 13 WRKY genes that were differentially expressed in IR28 and LYP9 after P1 inoculation (Fig. 6 and Additional file 11: Table S8). In IR28, five WRKY transcription factors were found to be significantly up-regulated. Among them, *OsWRKY53*, *OsWRKY69* and *OsWRKY71* genes were induced at both time points in IR28 and significantly inhibited at 48 hpi in LYP9, suggesting that these WRKY proteins might function as key positive regulators in the rice defense against the infection by *U. virens* during initial colonization.

### The *cis*-acting regulatory element analysis

Venn diagrams in Additional file 12: Figure S4 showed up-regulated and down-regulated DEGs with consistent expression patterns at both time points in IR28 and LYP9 in response to P1 inoculation . In IR28, 454 genes exhibited similar expression patterns between 24 hpi and 48 hpi, including 284 induced and 170 suppressed genes. In susceptible LYP9, 67 genes were up-regulated and 136 genes were down-regulated simultaneously at 24 hpi and 48 hpi. The conserved *cis*-elements in the promoter regions of the DEGs with similar expression patterns may provide clues as to how rice plants respond to pathogen infection. Eleven conserved motifs including five core elements of W-box and several DNA binding sites of Dof and Myb transcription factors were identified when comparing the promoter regions of up-regulated genes with those of down-regulated genes in IR28 (Additional file 13: Table S9). W-boxes, the binding sites of WRKY transcription factors, were significantly more abundant in the 5’-regulatory regions of up-regulated DEGs in IR28. Strikingly, *cis*-element scanning in the PLACE database revealed that a *cis*-element CTAGCTAG, where the RY repeat motif has been found to be essential for seed-specific expression of some storage proteins, was identified to be significantly more enriched in the promoter regions of up-regulated *PR* genes as compared to other *PR* genes in IR28 (Table 2). The *cis*-element is even more abundant in the promoters of the up-regulated chitinase gene cluster. For comparison, the frequency of the *cis*-element in the 52 up-regulated kinase gene promoters is similar to that in other coding genes in the genome. These data suggest that the RY repeat is a *cis*-regulatory motif that is involved in the regulation of defense-related genes.

**Table 2:** RY repeat motifs enriched in the 5’-regulatory regions of 47 *PR* genes, particularly in 9 chitinase genes, which were up-regulated in IR28 and suppressed in LYP9

Motif_seq	PR genes (47)			Chitinase (9)			PR genes (660)			Protein kinases (52)			Annotation	Motif_ID
	Num^a^	RAR^b^	*P*_value	Num	RAR	*P*_value	Num	RAR	*P*_value	Num	RAR	*P*_value		
CATGCATG	31	6.3387	8.89E-13	11	11.7459	3.85E-07	174	2.5336	8.03E-24	6	1.1089	0.8205	RY repeat motif	S000102
CATGCAT	55	5.1483	1.15E-15	16	7.8213	6.97E-07	337	2.2464	4.15E-31	14	1.1845	0.5290	RY repeat motif	S000105
CATGCAY	69	4.4198	3.05E-15	19	6.3557	2.10E-06	462	2.1074	2.07E-33	20	1.1579	0.5860	RY repeat motif	S000100
CATGCA	92	3.5564	2.15E-13	25	5.0468	7.38E-06	700	1.9270	6.24E-34	28	0.9783	1.0000	RY repeat motif	S000264

^a^The number of RY repeat motifs

^b^RAR = (motif count in a selected promoter set/number of promoters in the set)/(motif count in total promoters/number of total promoters)

*P* values were calculated using Fisher’s exact test

The total set of sequence-available *PR* genes (660) and that 52 protein kinase genes that were up-regulated in IR28 were used for comparisons

### Validation of DEGs by quantitative RT-PCR analyses

**Fig. 7:**
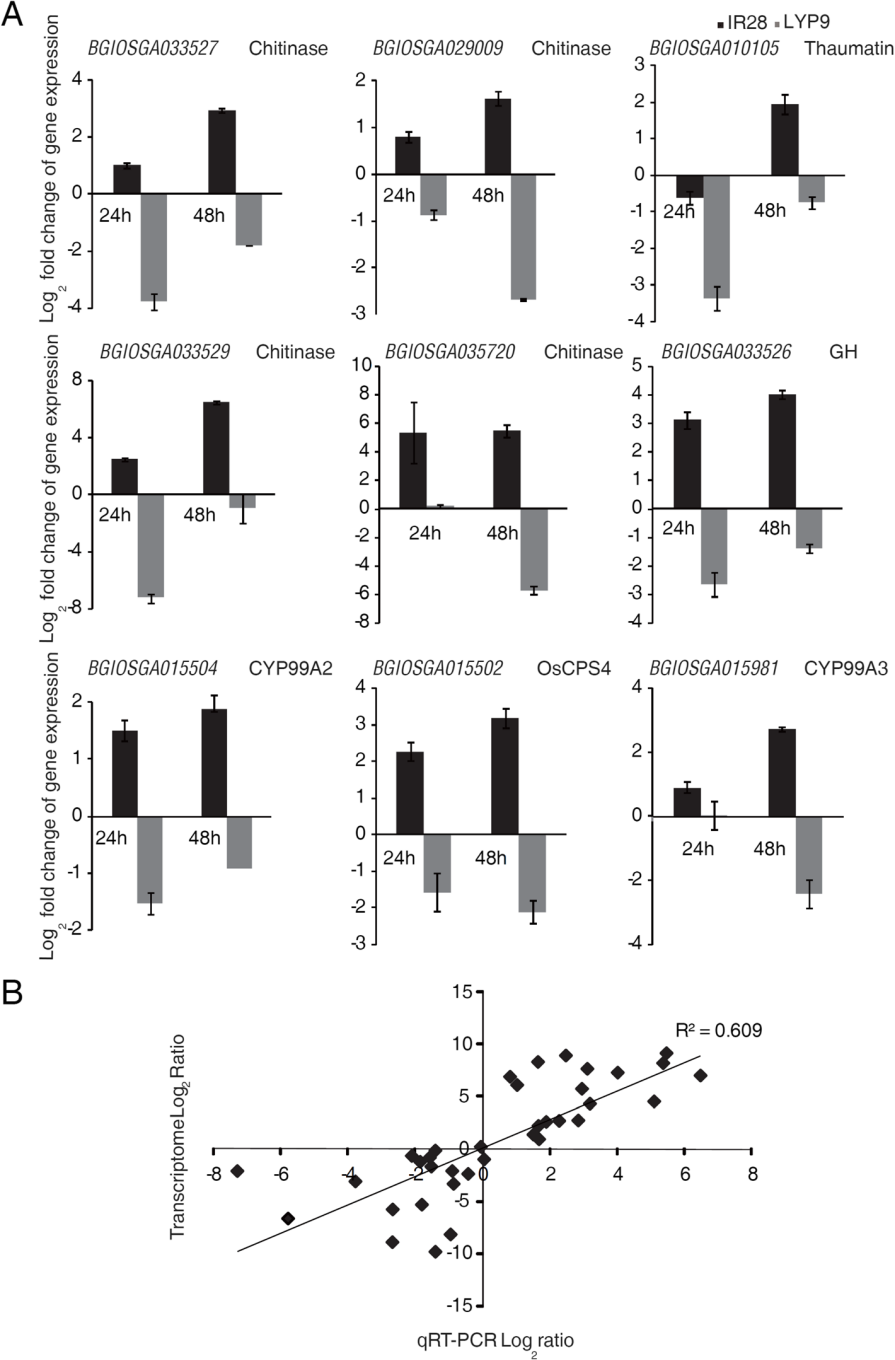
Time-course expression analyses and validation of nine selected DEGs using quantitative real time RT-PCR. **a** Quantitative RT-PCR analyses of nine selected DEGs confirmed that these genes were up-regulated in IR28 and generally suppressed in LYP9 at both 24 and 48 hpi. Log_2_ fold change of transcript levels in the inoculated samples with respect to the transcript levels in mock-inoculated rice panicles was shown. Error bars represent standard errors for three replicates of qRT-PCR assays. **b** The linear correlation between RNA-Seq transcriptome profiles and qRT-PCR data. The log_2_ ratio values from transcriptome data were plotted against those of the qRT-PCR results. A correlation coefficient of 0.61 indicates that there is a good linear correlation between RNA-Seq and qRT-PCR data

To validate the DEGs identified by comparative transcriptome analyses, 14 DEGs that might be essential for RFS resistance were selected, and expression of these genes in response to pathogen inoculation was investigated by quantitative real-time RT-PCR (qRT-PCR). The qRT-PCR results showed that most of the tested genes were generally up-regulated at 24 hpi and 48 hpi in the resistant IR28 and down-regulated in the susceptible LYP9 (Fig. 7a and Additional file 14: Figure S5). The correlation between RNA-Seq and qRT-PCR data was further validated by comparing the corresponding expression data from both analyses. The log_2_ ratio values from transcriptome analyses were plotted against those from qRT-PCR. A clear correlation between two methods was found at R^2^ = 0.61 (Fig. 7b). In general, qRT-PCR data confirm expression patterns of these important RFS resistance-related genes revealed by RNA-Seq analyses.

### Comparison of *U. virens* transcriptome in the resistant and susceptible cultivars during infection

To compare expression profiles of *U. virens* during infection of the resistant and susceptible cultivars, clean RNA-Seq reads were mapped to the reference genome of *U. virens* [13] (Additional file 15: Table S10). Expression profiles of *U. virens* from the infected resistant cultivar IR28 were analyzed and compared with those from LYP9 described previously [13]. In IR28, 614 and 542 fungal genes were up-regulated significantly at 24 and 48 hpi compared with that from axenic cultures, respectively. Meanwhile, 425 and 247 genes were identified to be suppressed at 24 and 48 hpi, respectively. Interestingly, predicted host-pathogen interaction database (PHI-base) genes [46] that are probably involved in host-pathogen interactions were found to be significantly enriched in fungal DEGs from both rice genotypes, indicating their potential roles in pathogenicity of *U. virens*.

As shown in Venn diagrams (Additional file 16: Figure S6), gene expression profiles of *U. virens* in the resistant cultivar IR28 were much different from those in the susceptible LYP9, although 426 (266 up-regulated and 160 down-regulated) and 433 (285 up-regulated and 148 down-regulated) genes have similar expression patterns during infection of the resistant and susceptible cultivars at 24 and 48 hpi, respectively. GO enrichment analyses revealed that *U. virens* DEGs in two cultivars, especially for down-regulated genes, were enriched in different GO terms (Additional file 17: Table S11). Interestingly, GO terms in biological processes that are related to fungal multiplication and pathogenicity, such as reproductive process, sexual and asexual reproduction, sporulation and cell adhesion, were significantly enriched in down-regulated genes in the resistant IR28, but not in the susceptible LYP9. These results suggest that biological processes required for successful infection of *U. virens* are greatly suppressed in the resistant cultivar.

## Discussion

RFS is a newly emerging fungal disease that causes severe yield loss and toxin contamination in rice grains [13]. Screening of rice genetic germplasm for RFS resistance revealed that certain cultivars exhibit relatively stable RFS resistance although no resistance gene has been reported so far. However, little is known about molecular mechanisms underlying durable resistance to RFS in rice. RNA-Seq is a recently developed approach that can be used in transcriptome analyses to reveal genome-wide expression profiling and regulation in plant hosts in response to pathogen infection. The technique has several advantages over other methods. First, RNA-Seq, unlike hybridization-based approaches, can detect gene transcripts despite not having the genome sequence of the target species. Second, RNA-Seq has low background noice [47]. Third, the technology has a higher sensitivity than DNA microarray and can be used to detect a larger dynamic range of expression levels of gene transcripts [48, 49].

In this study, RNA-Seq was used to identify genes differentially expressed between the cultivar IR28 with durable RFS resistance and susceptible cultivar LYP9 in response to *U. virens* at early infection stages. Comparative transcriptome analyses suggest that some important protein families including receptor-like kinases, WRKY transcription factors, PR proteins, and phytoalexin biosynthetic enzymes play important roles in RFS resistance. A clear correlation between RNA-Seq and qRT-PCR data confirmed expression patterns of the tested genes in response to *U. virens* infection (Fig. 7 and Additional file 16: Figure S6). Several transcriptome studies on the interaction of rice and *U. virens* have been reported recently [37, 38]. Different from other transcriptome analyses, we analyzed and compared transcriptome profiles of the resistant and susceptible rice cultivars at the very early stage of infection (24 hpi and 48 hpi). Although gene expression profiles were partially different among those studies, a large proportion of DEGs revealed here were also reported in other transcriptome analyses. For instance, WRKY transcription factors, such as WRKY53 and WRKY69, were induced in different transcriptome studies. Additionally, some genes that had unique responses to *U. virens* infection revealed by Chao et al. [37], such as LOC_Os07g07870.1 and LOC_Os08g23790.1, had similar expression patterns in this study. Difference in expression patterns of partial DEGs might be due to different infection stages and different rice genotypes. It has been found that many rice genes had opposite regulation patterns between the early and late stages of *U. virens* infection [38].

### Pathogenesis-related proteins may be crucial for RFS resistance

Cluster analyses showed that the majority of DEGs (inoculated vs. non-inoculated) in both genotypes were differentially regulated between the two cultivars in response to *U. virens* inoculation (Fig. 2). Among the group II genes, 47 *PR* genes were identified including members in the *PR*2-6, *PR*8 and *PR*9 families (Fig. 4). Some PR proteins, such as β-1,3-glucanases, chitinases and proteinases have direct antifungal activities and hydrolyze molecules on the cell walls of fungal pathogens, including glucans, chitins and proteins directly [50, 51]. Other PR proteins including thaumatin-like proteins and proteinase inhibitors have enzyme inhibitory activities and exert an effect against fungi by inactivating proteinases secreted by pathogens [52]. In addition, the peroxidase activity of PR-9 also contributes to fungal disease resistance by cross-linking and strengthening plant cell walls [53].

Consistent with our findings, *PR* genes in rice have been shown to be induced by diverse biotic stresses including infection by the rice blast fungus *M. oryzae* [36], the bacterial blight pathogen *Xanthomonas oryzae* pv. *oryzae* [54], the sheath blight fungus *Rhizoctonia solani* [55, 56], and the rice dwarf virus (RDV) [57]. These expression data suggest that *PR* genes have important roles in plant defenses against pathogen infection, which has been experimentally verified. Previous studies demonstrated that over-expression of the *PR* genes encoding β-1,3-glucanases, chitinases and thaumatin-like proteins enhanced resistance to *Fusarium* head blight in wheat [58–61].

Preliminary mapping using 157 recombinant inbred lines derived from an inter-subspecies cross of Daguandao/IR28 identified a QTL conferring RFS resistance in the chromosome 11 in IR28 [62]. The QTL is physically close to the chitinase gene cluster region, out of which, nine chitinase genes were identified to be highly induced after *U. virens* inoculation (Fig. 4 and Additional file 9: Table S6). Another study showed that a QTL conferring resistance to *R. solani* was also mapped near to the chitinase gene cluster region [63], suggesting that the chitinase gene cluster might be involved in broad-spectrum and durable disease resistance. Notably, clean RNA-Seq reads of the susceptible cultivar LYP9 were mapped to these chitinase genes and it was found that no gene in the chitinase cluster was absent from the genome of LYP9. Collectively, these differentially regulated *PR* genes in the resistant and susceptible genotypes might play essential roles in rice resistance against *U. virens.*

### Diterpene phytoalexins are important for RFS resistance

Diterpene phytoalexins, secondary metabolites with a low molecular mass, have anti-microbial activity and play important roles in plant defense responses [64, 65]. In this study, seven diterpene phytoalexin biosynthesis genes were identified to be significantly up-regulated in the resistant variety and weakly or not induced in the susceptible variety after inoculation (Fig. 5 and Additional file 10: Table S7). Among them, *OsCPS4, CYP99A2, CYP99A3* and *OsMAS* are responsible for different steps in the biosynthesis of momilactone A and B (Fig. 5). Knock-down of *OsCPS4* caused lower accumulation levels of momilactones and oryzalexin S and the *cps4* rice mutant is more susceptible to *M. oryzae* infection than the wild-type [66]. Simultaneous knock-down of *CYP99A2* and *CYP99A3* specifically suppressed elicitor-inducible production of momilactones [67]. Additionally, *OsCPS2* and *CYP76M7* are physically located on the same gene cluster involved in biosynthesis of the antifungal phytocassanes [68]. *OsCPS2* expression in the resistant rice cultivar IL7 was up-regulated at 2 d after *M. oryzae* inoculation, resulting in enhanced phytoalexin production [40]. *OsKSL11* is another gene where expression was elevated in IR28 after *U. virens* infection. OsKSL11 has been found to react with *syn-*CDP and produce *syn*-stemod-13(17)-ene [69]. These results suggest that production of phytoalexins, in particular momilactones, is highly induced by *U. virens* infection in rice and can play a key role in RFS resistance.

### Conserved *cis*-elements are involved in the regulation of defense responses against *U. virens* infection

A recent study reported that the *U. virens* regulated genes shared highly conserved *cis*-elements in the promoters including W-boxes, the DNA binding sites of Myb and Dof proteins, which is highly consistent with our *cis*-element enrichment analyses (Additional file 13: Table S9) [37]. WRKY transcription factors are vital components in plant defense against pathogens [70]. WRKY proteins can regulate phytoalexin production and *PR* gene expression through binding to the *cis*-regulatory element W-box. This study revealed that 13 WRKY transcription factors were differentially regulated in both the resistant and susceptible cultivars after *U. virens* infection. In particular, *OsWRKY53*, *OsWRKY69* and *OsWRKY71* were found to be highly up-regulated in IR28 and suppressed in LYP9 (Additional file 11: Table S8). It was demonstrated that transgenic rice plants over-expressing *OsWRKY53* and *OsWRKY71* exhibited enhanced resistance to blast disease and *X. oryzae* pv. *oryzae* infection [71–73]. Both Dof and Myb proteins are also important transcription factors that are involved in the regulation of plant defenses and biotic stress resistance [74, 75]. Taken together, these findings imply that some WRKY, Dof and Myb transcription factors, such as OsWRKY53, OsWRKY69 and OsWRKY71, play important roles in rice transcriptome regulation during *U. virens* infection.

Furthermore, the *cis*-regulatory RY repeat motif was found to be significantly more abundant in the promoter regions of these differentially regulated *PR* genes than other *PR* genes, even though the motif is generally enriched in the *PR* gene promoters. These results suggest that the seed-specific *cis*-element may be also involved in the expression regulation of defense-related genes in response to *U. virens* infection.

### Defense-oriented reprogramming of protein kinase genes in rice during early infection of *U. virens*

Many protein kinase genes were reported to be transcriptionally regulated in host plants upon pathogen infection [76]. In agreement with this, we found here that 52 protein kinase genes were highly induced in IR28 after *U. virens* infection. Among these, three categories of receptor-like kinases including lectin-, LRR- and LysM-containing transmembrane kinases were identified which are often involved in the recognition of pathogens by sensing pathogen-associated molecular patterns [77]. Many LysM receptor-like kinases can mediate plant defense responses against fungal pathogens likely through chitin perception [78, 79]. BGIOSGA016815, a lectin receptor kinase, was also identified to be induced in response to bacterial, parasite, fungal and viral infection in rice [80]. Other up-regulated kinase genes encode cytoplasmic kinases that function in the phospho-relay and are essential components in defense signaling. For instance, OsMAPKK4 is phosphorylated by upstream MAPKKK7 (BGIOSGA000957) that was induced by *U. virens* infection in IR28, which prompts signal transduction in response to various biotic and abiotic stresses including pathogen, insect, drought, salinity, flood and cold [81]. Therefore, we speculate that these differential regulated protein kinases may play crucial roles in RFS resistance signaling.

## Conclusion

In the present study, comparison of expression profiles between the resistant cultivar IR28 and the susceptible LYP9 during early stages of *U. virens* infection uncovered a clear difference in the regulation of defense responses against *U. virens* between the two genotypes. A genome-wide view of expression profiles of the resistant rice cultivar in response to *U. virens* infection promotes understanding of molecular mechanisms underlying RFS resistance. A specific set of protein kinases, PR proteins, WRKY transcription factors, and secondary metabolites including phytoalexins were found to be crucial for RFS resistance. Transgenic rice plants over-expressing some of the identified genes are being developed to confirm their biological functions in RFS resistance. The information revealed by transcriptome analyses will also facilitate the isolation of QTLs associated with resistance to *U. virens* in rice.

## Methods

### Rice materials and fungal inoculation

*Oryza sativa* L. spp. *indica* cultivars IR28 (resistant to RFS) and LYP9 (highly susceptible but high-yielding) were grown at the experiment station of Jiangsu Academy of Agricultural Sciences in Nanjing, Jiangsu, China. *U. virens* 37–1 and 39–3 were monospore isolates from samples collected at paddy fields in Jiangsu Province, China, and the P1 isolate originating from Kansas, USA was courtesy of Professor Jinrong Xu, Purdue University. Rice panicles were inoculated with a mixture of conidial and hyphal fragments as described with minor modifications [82]. Briefly, the *U. virens* isolates were cultured in potato sucrose broth (PSB, fresh potato extract and 2 % sucrose) on an incubator shaker at 120 rpm and 28 °C for a week. The panicles of rice plants at the booting stage were inoculated with conidial suspensions (2 × 10^5^ conidia ml^−1^) at 5 to 7 days before earing. Rice panicles injected with PSB were used as mock controls. The pathogen- or mock-inoculated panicles were harvested at 24 and 48 hpi, immediately frozen in liquid nitrogen, and then kept at −70 °C for RNA isolation. Some inoculated rice plants were grown further for disease symptom observations three weeks after inoculation.

### Preparation of cDNA libraries for RNA-Seq

Total RNA was isolated using RNApure® total RNA rapid extraction kit according to the manufacturer’s instruction (Aidlab Biotechnologies, Beijing). The yield and purity of RNA were evaluated by measurement of absorbance at 260 and 280 nm. RNA integrity was confirmed using Agilent 2100 Bioanalyzer (Agilent Technologies) with a minimum RNA integrated number (RIN) value of 7.0. Total RNA isolated from the samples of three biological replicates at each time point (24 and 48 hpi) was combined for RNA-Seq. Poly(A) + mRNA was enriched from total RNA using oligo(dT) magnetic beads and used for library construction. RNA-Seq libraries were constructed following the standard pipeline at Beijing Genomics Institute (BGI) in Shenzhen, China. Reads of 49 bp length were generated with the Illumina HiSeq™ 2000 sequencing platform at BGI.

### Mapping reads to the reference genome and annotated genes

Raw reads were downloaded from BGI in FASTQ format. The reference genome of *Oryza sativa* L. ssp. *indica* 93*–*11 and associated gene information were downloaded from Gramene (http://www.gramene.org/) and the Rice Genome Annotation Project (http://rice.plantbiology.msu.edu). The genome of *U. virens* isolate UV-8b was used as the reference for analyzing *U. virens* transcriptome [13]. Prior to mapping reads to the reference databases, all reads were filtered to remove adaptor sequences, and eliminate reads in which the percentage of unknown bases (N) was greater than 10 %, or the percentage of the low quality bases (bases with Phred quality score ≤ 5) in a read exceeded 50 %. The resultant clean reads were mapped to rice and *U. virens* genomes using SOAP2 [83]. No more than two mismatches were allowed in the alignment for each read.

### Analysis and screening of differentially expressed genes 

RPKM (Reads per kb per Million reads) was used to represent the gene expression level of rice and *U. virens* transcripts [48]. Differentially expressed genes (DEGs) in rice cultivars were identified through comparing gene expression levels between *U. virens-* and mock-inoculated panicles with the criteria of the absolute log_2_ ratio value ≥ 1 and false discovery rate (FDR) ≤ 0.001 [84]. DEGs of *U. virens* were identified by comparing the gene expression level during infection with that in axenic cultures using the same criteria. The DEGs of rice and *U. virens* were then subjected to GO enrichment analyses using the WEGO (Web Gene Ontology Annotation Plotting) program, respectively [85]. *P*-values were calculated by comparing the observed frequency of an annotation term with the frequency expected in respective genome using Pearson’s chi-squared test. The Kyoto Encyclopedia of Genes and Genomes (KEGG) pathway enrichment analysis was performed to identify significantly enriched metabolic pathways or signal transduction pathways in rice DEGs comparing with the whole genome background. Pathways with Q-values ≤ 0.05 are considered significantly enriched in DEGs as assessed with the PAICE program [86]. Hierarchical clustering of all DEGs was performed using cluster 3.0 [87].

### Conserved *cis*-elements searches

The 1.5 kb sequences upstream of the start codon of selected genes in rice were scanned for putative conserved *cis*-elements identical with or similar to the motifs in PLACE database [88]. The enriched motifs in the up-regulated genes were determined by comparing frequency in the up-regulated genes with that in down-regulated genes (chi-square test, *P* < 0.01). Alternatively, Relative Appearance Ratio (RAR) of motifs was calculated using the formula (motif counts in a selected promoter set/number of promoters in the set)/(motif counts in total promoters/number of total promoters) [89]. *P* values comparing motif frequency in selected gene sets with that in total genes were calculated using Fisher’s exact test. The conserved motifs were identified with the criteria of RAR ≥ 3 and *P* value < 0.01.

### Validation of RNA-Seq data by quantitative real-time RT-PCR

Some differentially regulated genes identified through RNA-Seq were validated by qRT-PCR. The primer sets used for qRT-PCR were designed based on exon sequences of the selected genes using the online program, oligo analyzer (http://www.idtdna.com) and the specificity of PCR primers was evaluated by blasting primer sequences against the NCBI database (Additional file 18: Table S12). Total RNA (2 μg) was used for cDNA synthesis with MLV reverse transcriptase (Invitrogen). PCR was performed in 20 μl of reaction mix containing 0.4 μl cDNA, 10 μl SYBR Premix Ex Taq™ (Takara, Dalian), 0.4 μl ROX reference dye, and 0.4 μl of each primer (10 μM) using an ABI Prism 7000 System (Applied Biosystems, Foster City, CA). Three replicates for each biological replicate were performed with similar results. Relative gene expression was calculated using the 2^-▵▵Ct^ method [90].

## Additional files

Additional file 1: Figure S1. Disease symptoms observed on LYP9 and IR28 panicles three weeks after *U. virens* P1 inoculation. False smut balls formed on a representative LYP9 panicle (**A**) and on a representative IR28 panicle (**B**) after P1 inoculation. The number of false smut balls formed on LYP9 panicles was significantly more than that on IR28 panicles. (TIFF 21 kb) Additional file 2: Table S1. Statistics of the total RNA-Seq reads and reads mapped to the *Oryza sativa* L. ssp. *indica* reference genome at different inoculation time points in IR28 and LYP9. (XLSX 21 kb) Additional file 3: Figure S2. Saturation analyses of RNA-Seq data. The number of detected genes approached saturation when RNA sequencing reads reached ~3 million for each library. (TIFF 21 kb) Additional file 4: Table S2. The differentially expressed genes that exhibited opposite expression patterns between IR28 and LYP9 at 24 hpi and 48 hpi. (XLSX 21 kb) Additional file 5: Table S3. The enriched GO terms for the genes that were up-regulated in IR28 and LYP9 at 24 hpi and 48 hpi. (XLSX 21 kb) Additional file 6: Figure S3. The expression pattern of DEGs in the enriched GO term “transferase activity” (GO: 0016740) specific to IR28. A total of 142 transferase genes were identified to be differentially regulated in IR28 in response to *U. virens* infection at 24 and 48 hpi. Black dots indicate the transferase genes encoding protein kinases. Each column represents the Log_2_ fold change in gene transcript levels in rice at the indicated times, relative to the levels of mock-inoculated samples. The vertical dimension represents the transferase genes that exhibited changes in transcript level (cutoff: |log_2_[fold change]| ≥ 1 and FDR ≤ 0.001). The colour scale indicates transcript abundance relative to the mock-inoculated panicles: red, increase in relative transcript abundance; blue, decrease in relative transcript abundance. (TIFF 21 kb) Additional file 7: Table S4. Protein kinase genes in the GO term “transferase activity” (GO: 0016740) that were induced by *U. virens* in IR28 and repressed in LYP9. (XLSX 21 kb) Additional file 8: Table S5. The enriched signaling and biosynthetic pathways of DEGs in IR28 and LYP9 at 24 hpi and 48 hpi revealed by KEGG enrichment analysis. (XLSX 21 kb) Additional file 9: Table S6. Differentially expressed *PR* genes between IR28 and LYP9 at 24 h and 48 h after *U. virens* infection. (XLSX 21 kb) Additional file 10: Table S7. Differential expression of phytoalexin synthesis genes in the susceptible variety LYP9 and the resistant variety IR28 at the early infection stages of *U. virens* (24 hpi and 48 hpi). (XLSX 21 kb) Additional file 11: Table S8. Differentially expressed WRKY genes in IR28 and LYP9 at 24 h and 48 h after *U. virens* infection. (XLSX 22 kb) Additional file 12: Figure S4. Venn diagrams showing the number of genes that exhibited similar expression patterns between 24 hpi and 48 hpi in IR28 (R) and in LYP9 (S). (TIFF 21 kb) Additional file 13: Table S9. The conserved motifs that were more abundant in the 5’-regulatory regions of up-regulated genes compared with down-regulated genes in IR28. (XLSX 21 kb) Additional file 14: Figure S5. Quantitative real-time RT-PCR analyses of six more differentially regulated genes in rice. The results showed that these genes were generally up-regulated in IR28 and suppressed in LYP9 at both 24 and 48 hpi, which is well consistent with transcriptome data. Log_2_ fold change of transcript levels in the inoculated samples with respect to the transcript levels in mock-inoculated rice panicles was shown. Error bars represent standard errors for three replicates of qRT-PCR assays. (TIFF 21 kb) Additional file 15: Table S10. Statistics of RNA-Seq reads mapped to the reference genome of *U. virens* at 24 h and 48 h after inoculation in IR28 and LYP9. (XLSX 21 kb) Additional file 16: Figure S6. Venn diagrams of differentially regulated *U. virens* genes in IR28 (R) and in LYP9 (S) at 24 hpi and 48 hpi. The majority of up-regulated (**A**) and down-regulated genes (**B**) exhibited different expression patterns in IR28 (R) and LYP9 (S) although a large proportion of DEGs shared the similar regulation patterns. (TIFF 21 kb) Additional file 17: Table S11. The enriched GO terms under three different categories for up-regulated and down-regulated *U. virens* genes in IR28 and LYP9. (XLSX 21 kb) Additional file 18: Table S12. The primers designed for quantitative real-time RT-PCR. For each gene, the forward (F) and reverse (R) primer sequences were listed. (XLSX 21 kb)
