# Supplementary figures and images for: Differential expression profiling of the early response to Ustilaginoidea virens between false smut resistant and susceptible rice varieties
Source: BMC Genomics. 2015 Nov 16;16:955. doi: 10.1186/s12864-015-2193-x (PMC4647755; doi:10.1186/s12864-015-2193-x)

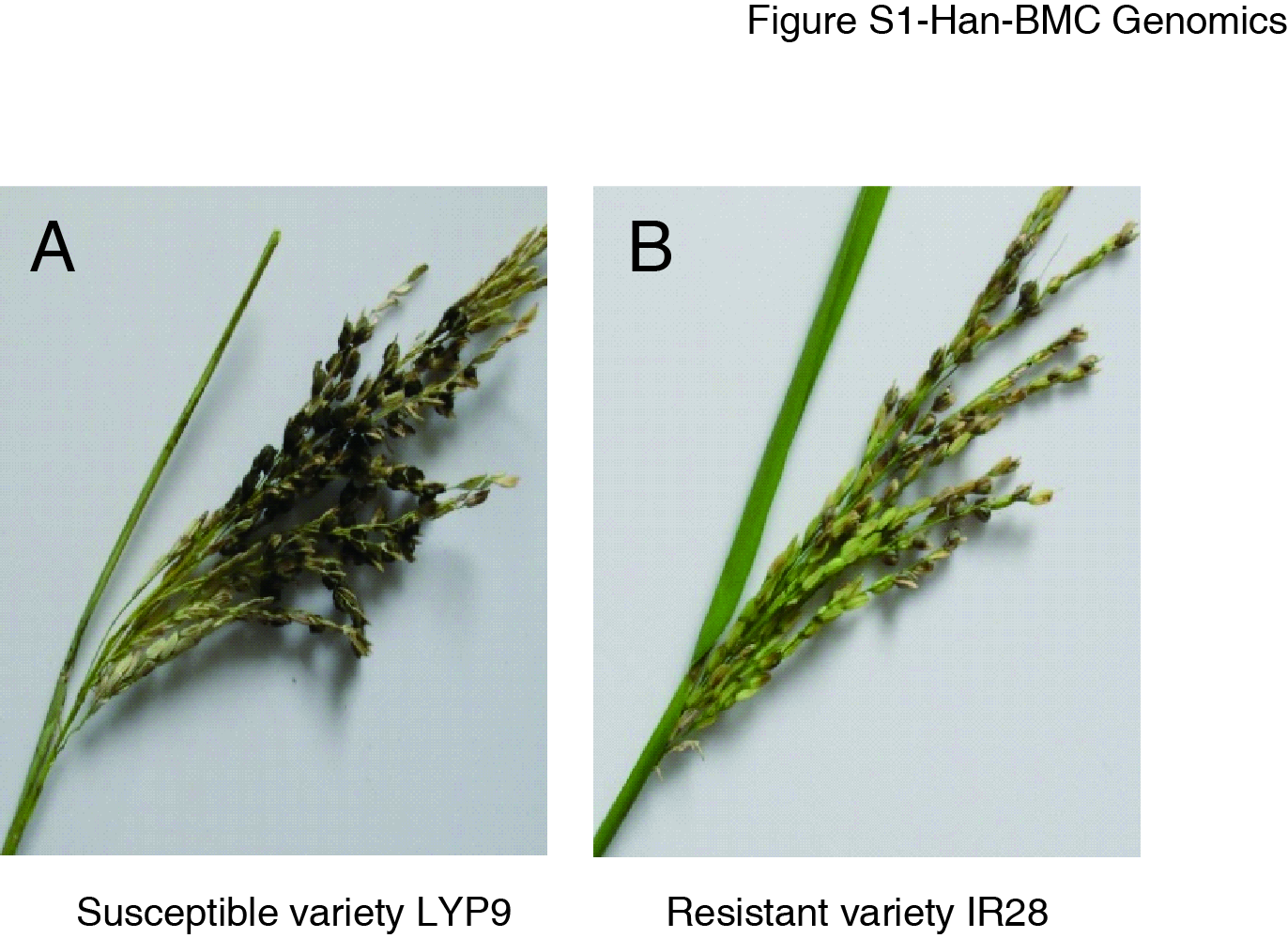

Supplement: Additional file 1: Figure S1. — Disease symptoms observed on LYP9 and IR28 panicles three weeks after U. virens P1 inoculation. False smut balls formed on a representative LYP9 panicle (A) and on a representative IR28 panicle (B) after P1 inoculation. The number of false smut balls formed on LYP9 panicles was significantly more than that on IR28 panicles. (TIFF 21 kb) [file 12864_2015_2193_MOESM1_ESM.tiff]

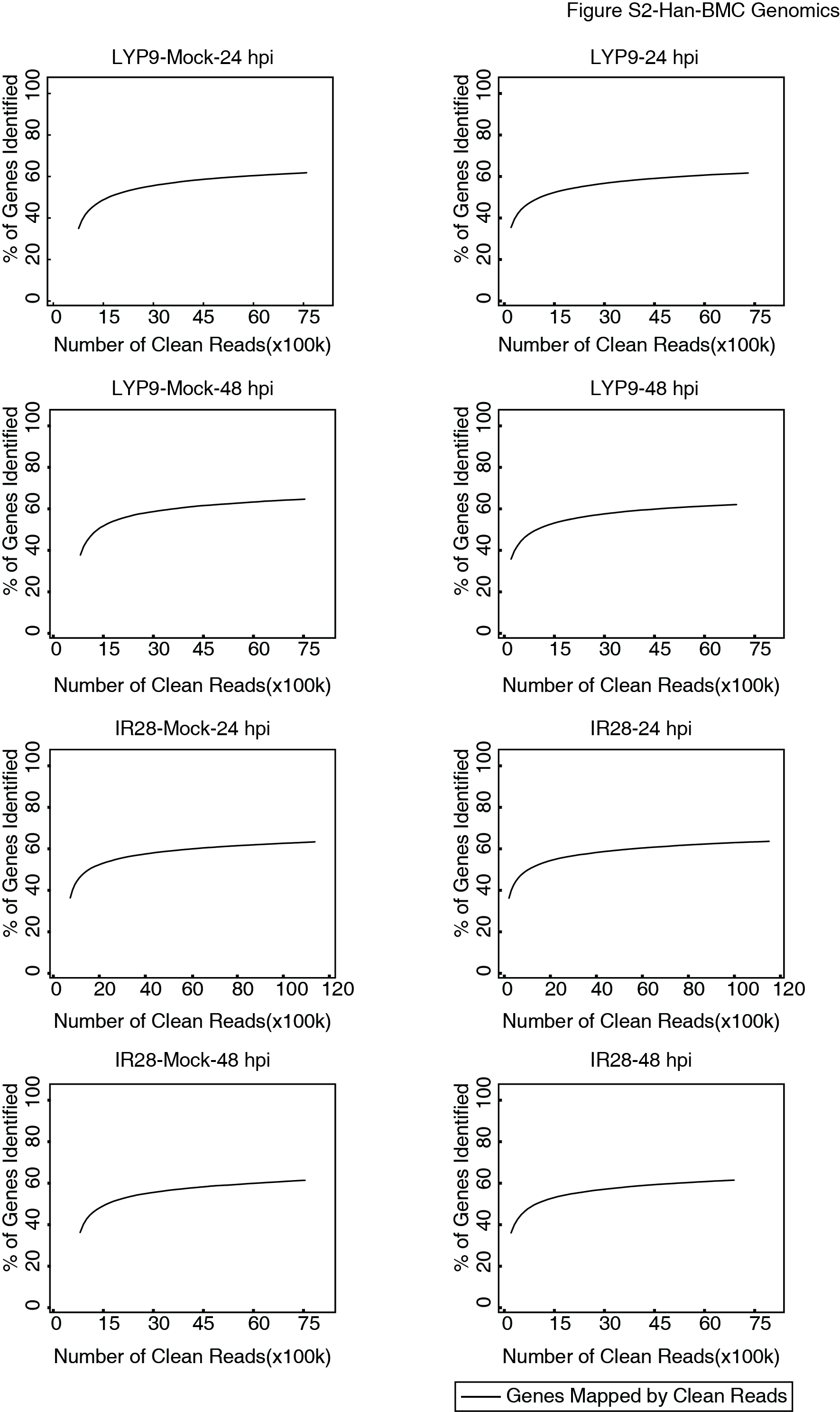

Supplement: Additional file 3: Figure S2. — Saturation analyses of RNA-Seq data. The number of detected genes approached saturation when RNA sequencing reads reached ~3 million for each library. (TIFF 21 kb) [file 12864_2015_2193_MOESM3_ESM.tiff]

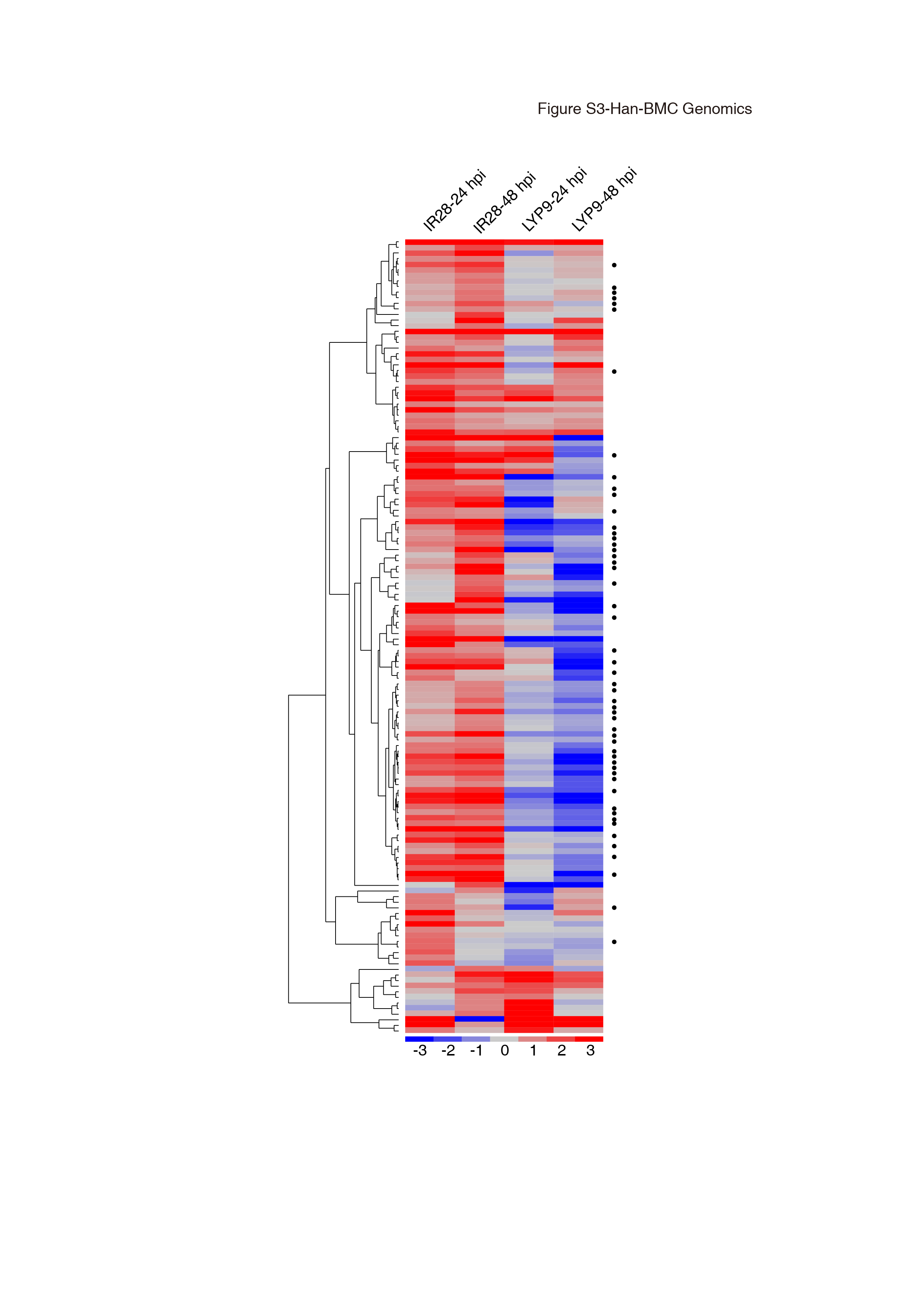

Supplement: Additional file 6: Figure S3. — The expression pattern of DEGs in the enriched GO term “transferase activity” (GO: 0016740) specific to IR28. A total of 142 transferase genes were identified to be differentially regulated in IR28 in response to U. virens infection at 24 and 48 hpi. Black dots indicate the transferase genes encoding protein kinases. Each column represents the Log2 fold change in gene transcript levels in rice at the indicated times, relative to the levels of mock-inoculated samples. The vertical dimension represents the transferase genes that exhibited changes in transcript level (cutoff: |log2[fold change]| ≥ 1 and FDR ≤ 0.001). The colour scale indicates transcript abundance relative to the mock-inoculated panicles: red, increase in relative transcript abundance; blue, decrease in relative transcript abundance. (TIFF 21 kb) [file 12864_2015_2193_MOESM6_ESM.tiff]

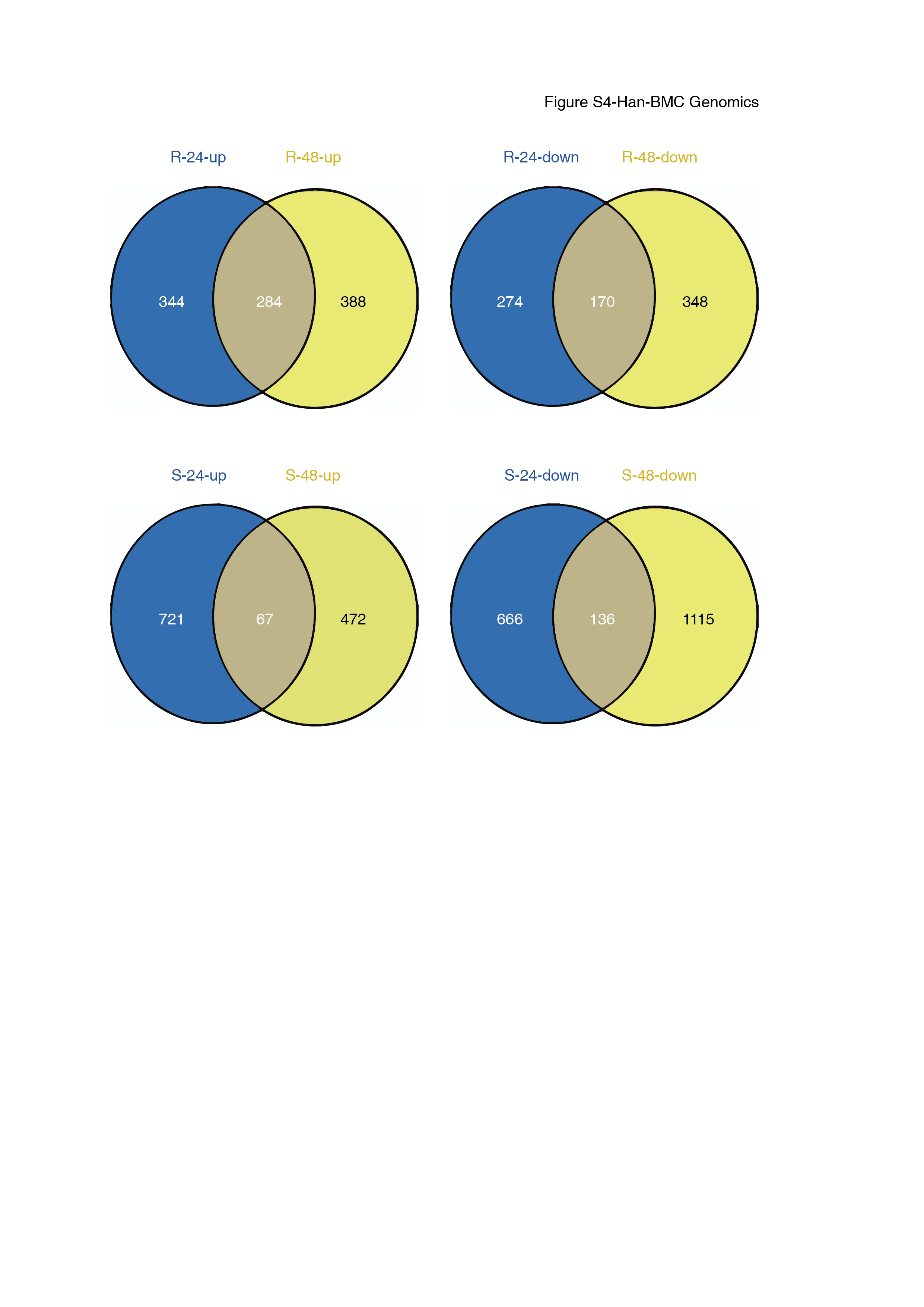

Supplement: Additional file 12: Figure S4. — Venn diagrams showing the number of genes that exhibited similar expression patterns between 24 hpi and 48 hpi in IR28 (R) and in LYP9 (S). (TIFF 21 kb) [file 12864_2015_2193_MOESM12_ESM.tiff]

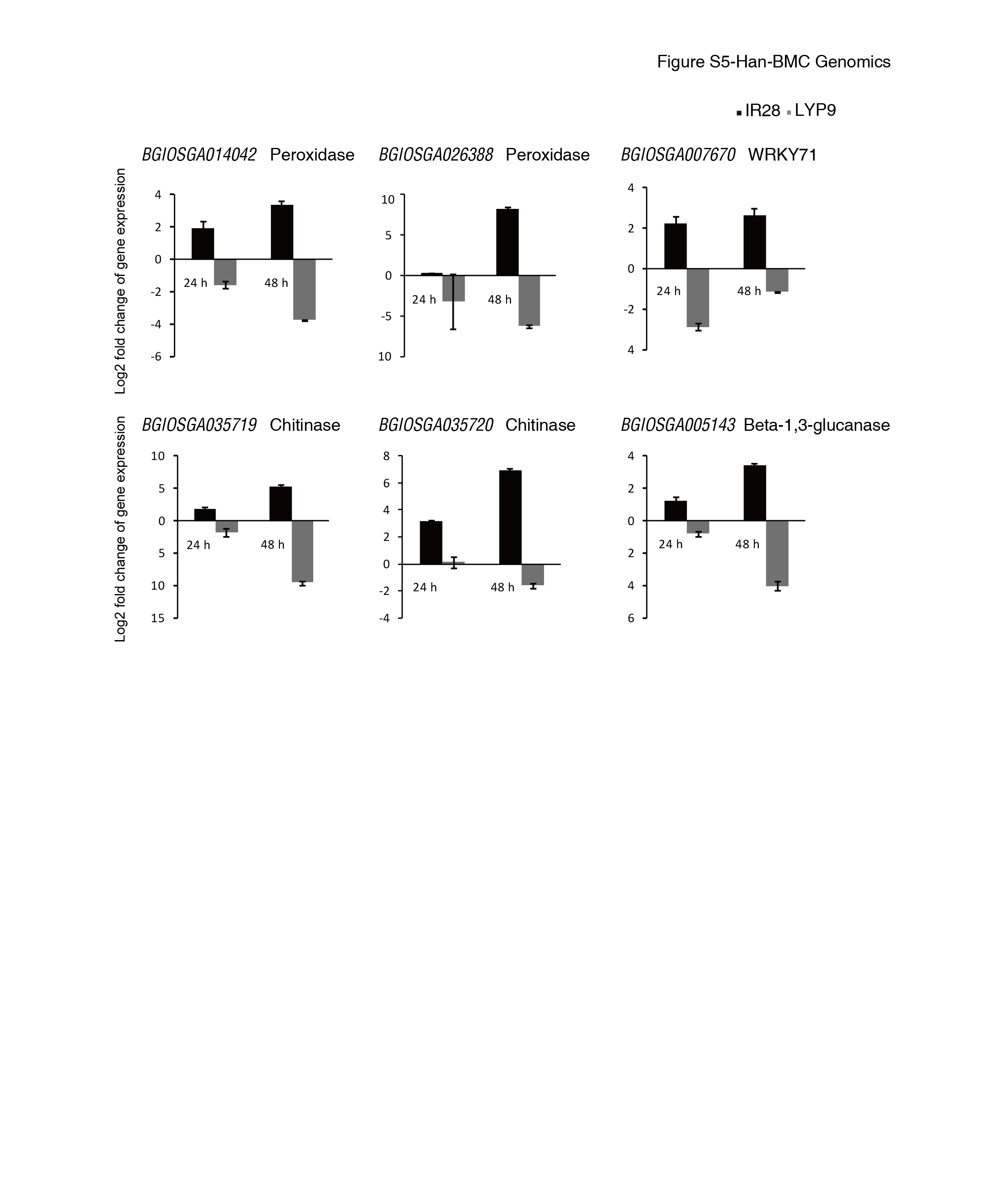

Supplement: Additional file 14: Figure S5. — Quantitative real-time RT-PCR analyses of six more differentially regulated genes in rice. The results showed that these genes were generally up-regulated in IR28 and suppressed in LYP9 at both 24 and 48 hpi, which is well consistent with transcriptome data. Log2 fold change of transcript levels in the inoculated samples with respect to the transcript levels in mock-inoculated rice panicles was shown. Error bars represent standard errors for three replicates of qRT-PCR assays. (TIFF 21 kb) [file 12864_2015_2193_MOESM14_ESM.tiff]

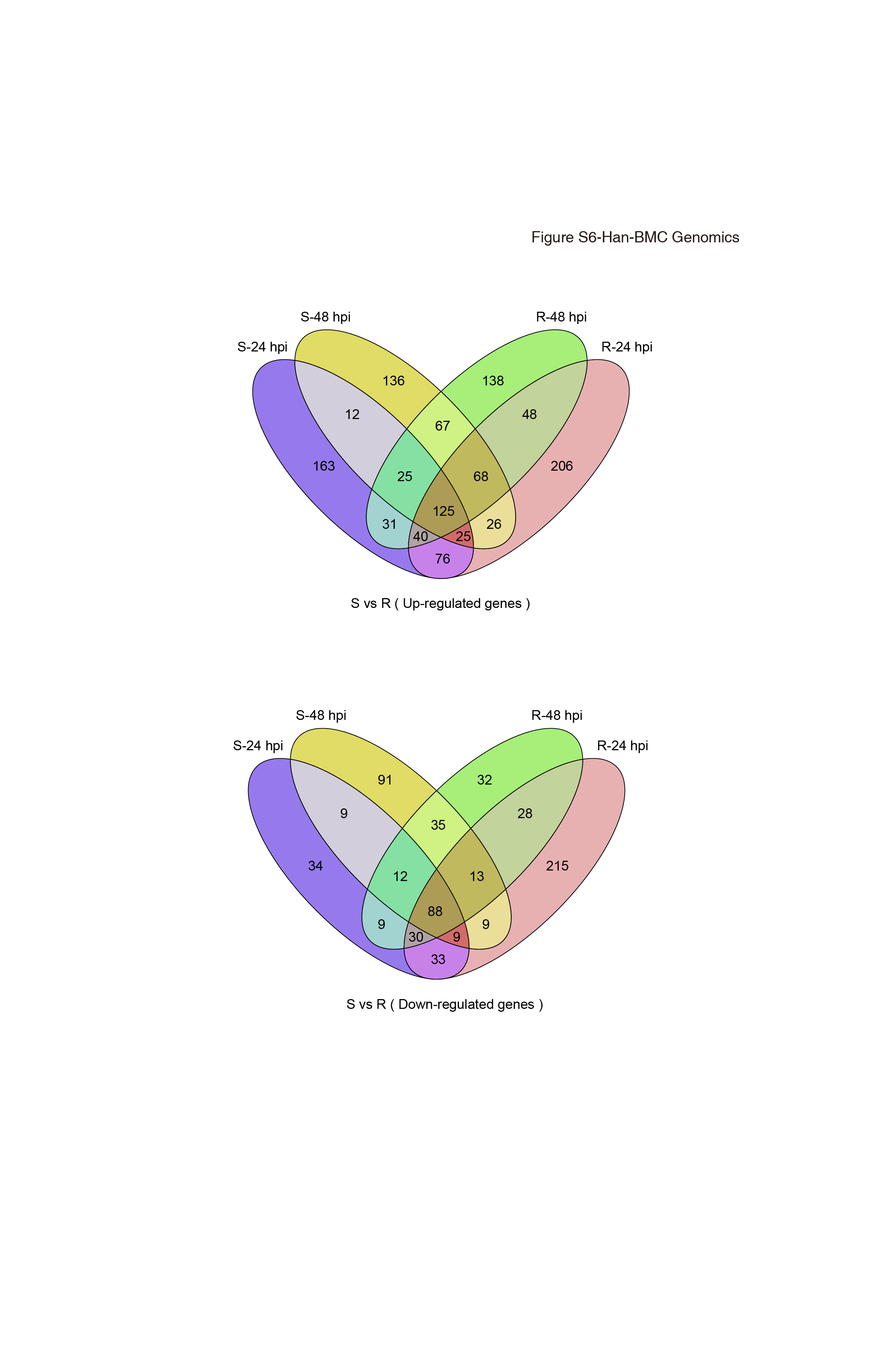

Supplement: Additional file 16: Figure S6. — Venn diagrams of differentially regulated U. virens genes in IR28 (R) and in LYP9 (S) at 24 hpi and 48 hpi. The majority of up-regulated (A) and down-regulated genes (B) exhibited different expression patterns in IR28 (R) and LYP9 (S) although a large proportion of DEGs shared the similar regulation patterns. (TIFF 21 kb) [file 12864_2015_2193_MOESM16_ESM.tiff]
